# Nutrients, Mitochondrial Function, and Perinatal Health

**DOI:** 10.3390/nu12072166

**Published:** 2020-07-21

**Authors:** Ameyalli M Rodríguez-Cano, Claudia C Calzada-Mendoza, Guadalupe Estrada-Gutierrez, Jonatan A Mendoza-Ortega, Otilia Perichart-Perera

**Affiliations:** 1 Section for Postgraduate Studies and Research, Higher School of Medicine, Instituto Politécnico Nacional, Mexico City 11340, Mexico; rocameyalli@gmail.com (A.M.R.-C.); ccalzada@ipn.mx (C.C.C.-M.); 2 Nutrition and Bioprogramming Department, Instituto Nacional de Perinatología Isidro Espinosa de los Reyes, Montes Urales 800, Lomas de Virreyes, Mexico City 11000, Mexico; 3 Instituto Nacional de Perinatología Isidro Espinosa de los Reyes, Research Division; Montes Urales 800, Lomas de Virreyes, Mexico City 11000, Mexico; gpestrad@gmail.com; 4 Immunobiochemistry Department, Instituto Nacional de Perinatología Isidro Espinosa de los Reyes, Montes Urales 800, Lomas de Virreyes, Mexico City 11000, Mexico; jonatan.mdz93@gmail.com; 5 Immunology Department, National School of Biological Sciences, Instituto Politécnico Nacional, Mexico City 11350, Mexico

**Keywords:** micronutrients, macronutrients, vitamins, minerals, diet, pregnancy

## Abstract

Mitochondria are active independent organelles that not only meet the cellular energy requirement but also regulate central cellular activities. Mitochondria can play a critical role in physiological adaptations during pregnancy. Differences in mitochondrial function have been found between healthy and complicated pregnancies. Pregnancy signifies increased nutritional requirements to support fetal growth and the metabolism of maternal and fetal tissues. Nutrient availability regulates mitochondrial metabolism, where excessive macronutrient supply could lead to oxidative stress and contribute to mitochondrial dysfunction, while micronutrients are essential elements for optimal mitochondrial processes, as cofactors in energy metabolism and/or as antioxidants. Inadequate macronutrient and micronutrient consumption can result in adverse pregnancy outcomes, possibly through mitochondrial dysfunction, by impairing energy supply, one-carbon metabolism, biosynthetic pathways, and the availability of metabolic co-factors which modulate the epigenetic processes capable of establishing significant short- and long-term effects on infant health. Here, we review the importance of macronutrients and micronutrients on mitochondrial function and its influence on maternal and infant health.

## 1. Introduction

Nutrition in early life is especially important, as the plasticity of developing organs defines how the organism reacts to challenges later in life. Intrauterine nutrient imbalances can cause changes in gene expression, which may alter the structure and function of certain organs in the offspring [1,2,3]. Maternal nutrition during pregnancy, including malnutrition and excess nutrients, has emerged as a critical risk factor for a number of non-communicable diseases (NCD) such as obesity, diabetes, hypertension, cardiovascular disease, non-alcoholic fatty liver disease, and neurocognitive disorders, among others. NCDs are the leading cause of death worldwide, and, although they are generally preventable, the currently existing strategies are insufficient [1,4].

Pregnancy entails a challenge for the maternal body systems, due to profound anatomical, physiological, and biochemical adaptations. These changes begin shortly after fertilization and continue through gestation, as a response to the physiological stimuli from the fetus and the placenta [5]. Mitochondria are the most abundant organelle in the oocyte and experience substantial structural and positional changes during preimplantation development [6]. As the main energy powerhouse, mitochondria are critical to maternal metabolism, on which fetal growth and development depend [6,7]. The objective of this work is to review the role of macronutrients and micronutrients on mitochondrial function and its influence on maternal and infant health.

## 2. Mitochondrial Function

Mitochondria are independent organelles which meet the energy requirement of the cell by producing adenosine triphosphate (ATP) through oxidative phosphorylation (OXPHOS) [7,8,9]. Mitochondria have their own DNA (mtDNA), which encodes for many of the essential components of the OXPHOS chain, and, thus, is crucial to ATP generation [10,11]. Mitochondria are essential to cellular and metabolic homeostasis and play major roles in both physiological and pathological processes. Although mitochondria have traditionally been seen only as passive signaling effectors, increasing evidence has indicated their active involvement in different pathways, as signal initiators and transducers through the modulation of metabolite availability and changes in the redox state [6]. They have an important role in regulating multiple cellular functions, including cell proliferation, apoptosis, innate immunity, inflammation, autophagy, redox signaling, calcium homeostasis, and stem cell reprogramming [11,12]. The mitochondrial matrix houses numerous metabolic pathways such as fatty acid oxidation, the tricarboxylic acid (TCA) cycle, and the synthesis of lipids and cholesterol [13]. They contribute to important functions such as the oxidation of pyruvate and fatty acids, the urea cycle, and the metabolism of amino acids and iron [5,14,15].

Important metabolites are produced by mitochondria, as derived from TCA cycle, including adenine β-nicotinamide dinucleotide (NAD+), α-ketoglutarate (α–KG), acetyl coenzyme A (AcCoA), and the further production of ATP, which are necessary co-substrates for several transcriptional and epigenetic processes (e.g., chromatin remodeling, histone modifications, and nucleosome positioning) [15]. In addition to the direct provision of substrates, mitochondria can indirectly impact on epigenetic signaling through the generation of reactive oxygen species (ROS) [15]. Mitochondria are the main source of ROS, a by-product of ATP production formed through electron leakage from OXPHOS [11,16,17]. Consequently, ROS-mediated epigenetic alterations may lead to altered expression of genes that regulate mitochondrial metabolism [15]. Due to their proximity to the electron transport chain (ETC)—the main ROS production site—mitochondrial proteins and mtDNA are vulnerable to oxidative damage, affecting the integrity of the mtDNA, membrane potential, calcium transport, inhibiting complexes in the ETC, and activating proapoptotic signals [15].

Under physiological conditions, ROS are involved in various cellular activities, including the activation of redox-sensitive transcription factors and protein kinases, regulation of vascular tone and functions controlled by oxygen concentrations, enhancement of signal transduction from many membrane receptors, and response against pathogens, among others [18]. However, in a situation of chronic production of ROS, their neutralization capacity is exceeded, leading to oxidative stress [16,19]. Oxidative stress in mitochondria decreases ATP production due to direct inhibitory effects on ETC complexes [20]. The normal response of the cell to the production of ROS is mediated through endogenous antioxidant systems, such as glutathione peroxidase (GPX), catalase, and superoxide dismutase (SOD), which are the main enzymes involved in the conservation of redox homeostasis, SOD being essential for the survival of aerobic organisms [16,21]. These proteins are abundant in most cells; they can hunt ROS by accepting electrons and becoming oxidized, although they are recycled by donating their electrons to acceptor molecules such as reduced nicotinamide adenine dinucleotide (NADH). The metabolic performance of the cell is correlated with its antioxidant response, and NADH levels are critical to the activity of many antioxidants [16]. Other substances, such as vitamin C, vitamin E, and glutathione (GSH), also have antioxidant functions [22].

Mitochondrial activities have been related to their morphology, which is determined by the ongoing processes of fusion and fission of their outer and inner membranes. The mitochondrial morphology is constantly adjusted to counteract metabolic insults, but also by signaling pathways; this allows compensatory changes in mitochondrial function to meet cellular energy and metabolic demands. Healthy mitochondria are maintained through multiple processes, including fusion (union of mitochondria resulting in a hyperfused network), fission (division leading to mitochondrial fragmentation), biogenesis (de novo formation), and mitophagy (removal of damaged mitochondria). The balance between these processes regulates their number, size, stability, distribution, and mitochondrial quality [10,12,23,24,25].

## 3. Mitochondrial Function in Pregnancy

Mitochondria seem to play a critical role in the physiological adaptations experienced during pregnancy. Their dynamic nature is crucial in modulating central cellular activities, turning into the main source of maternal metabolic energy for fetal development and matching the embryo’s changing energy requirements [6,7]. Additionally, they regulate processes such as folliculogenesis, oocyte maturation, corpus luteum and uterine function, embryogenesis, embryonic implantation, and fetoplacental development, through ROS signaling at physiological levels [8].

Placenta requires ATP for active cell transport, mainly from aerobic cellular respiration. However, glucose is transported across the placenta by facilitated diffusion. This could imply that decreased fetal growth could be a result of impaired mitochondrial function leading to insufficient placental ATP production, consequently leading to placental insufficiency [26] (the inability of the placenta to supply the nutrients and oxygen required by the fetus) and hypoxia (as impaired maternal blood flow leads to low oxygen) [27,28]. If placental ATP production is restricted, fetal growth may be affected, as energy for nutrient and oxygen transport would be limited [26].

Adequate control of mitochondrial dynamics leads to an appropriate mtDNA count, which is necessary to regulate embryonic development and implantation potential, as well as fetal and placental growth, ensuring optimal regulation of chromosome segregation [6,7]. Mitochondrial location also changes considerably throughout the development of the early embryo [6]. This mitochondrial trafficking transports the mitochondria to high energy demand sites (e.g., in pronuclear oocytes and early cleavage stage embryos, during the blastocyst stage, the embryo exhibits high levels of both glycolysis and oxygen (O2) consumption [29,30,31]), facilitates communication and interaction with other organelles to support embryonic development, and maintains metabolites and signaling gradients with the nucleus. Adequate communication between the mitochondria, the nucleus, and other organelles promotes cellular homeostasis in the developing embryo. Failure or misregulation of these interactions can adversely affect the embryo’s ability to respond to stress and regulate mitochondrial function, or lead to a modification of the epigenetic scene, in response to changes in nutrient availability, affecting viability and long-term health. Nutrient availability could disturb this balance [6].

Few human studies have reported results on mitochondrial function during healthy pregnancy, showing adaptations of mitochondria when compared to non-pregnant women [5,7]. On the other hand, some studies have documented that oxidative stress increases with gestational age in healthy pregnancies [32,33]. At the beginning of pregnancy, placental O_2_ is relatively low, which is essential for normal cell proliferation and placental angiogenesis; such low O_2_ could protect the embryo from ROS at that time. By the end of the first trimester, once maternal intraplacental circulation is fully established, the O_2_ tension triples along with ROS levels. The placenta then adapts to this increase by modulating hypoxia-inducible factor 1α (HIF-1α) and increasing cellular antioxidant levels [18]; the placenta is armed with antioxidant defenses, which protect it from any undue harm [34]. GPX and SOD activities are increased in the third trimester of normal pregnancies, with positive correlations with markers of oxidative stress [32]. Under normal conditions, these adaptations are favorable to fetal development [18].

Adaptations in placental mitochondrial function have been documented in pregnant women with high oxidative stress [35]. Oxidative stress has been linked with several pregnancy complications, including miscarriages, embryopathies, premature delivery, and intrauterine growth restriction (IUGR) associated with pre-eclampsia (PE). Some possible mechanisms include excessive ROS production due to a reduced activity of the antioxidant system, which may cause changes in mtDNA copy number and mitochondrial function [21]. It has been hypothesized that, during pregnancy under oxidative stress, the activity of relevant transcription factors that stimulate mitochondrial biogenesis is promoted, in order to ensure its preservation [5,8]. With increasing maternal adiposity, there is a significantly increased generation of ROS, reduced expression levels of subunits of complexes I–V, and decreases in mitochondrial respiration by OXPHOS and in ATP generation in the placenta [36]. In contrast, total antioxidant capacity and SOD activity are significantly greater in the placentas of normal weight mothers than placentas from obese mothers [37]. Furthermore, catalase activity and GPX activity are decreased in pre-term placentas, compared to term placentas [38]. Normoglycemic obese pregnant women could have increased mitochondrial biogenesis, when compared to normal-weight pregnant women [39].

IUGR and PE are disorders characterized by defective placental function, leading to poor transfer of oxygen and nutrients to the fetus, elevated inflammation, and oxidative stress. Mitochondria could potentially be associated with the pathogenesis of placental insufficiency. The quantity of mitochondria mirrors the energy requirement of the cells; the number of mitochondria can be altered under oxidative stress or hypoxic and nutrient-deprived environments, affecting mitochondrial function [40].

Some studies have documented changes in mitochondrial function in the face of pregnancy complications. In pre-eclamptic pregnancies, compared to non-complicated ones, ATP levels are decreased [41] and citrate synthase activity (which relates to mitochondrial function/content) is increased [42]. Mitochondrial SOD activity is reduced in pre-term pre-eclamptic placentae, compared to pre-term controls [38]. Furthermore, mitochondrial fusion/fission seems to be impaired in PE, although this relationship is complex and still poorly understood [43]; some findings have indicated a pro-fusion state in severe cases of PE [42], while other research has shown a more pro-fission state [41,44]. Additionally, there is a decrease in TFAM (Mitochondrial Transcription Factor A) expression [42] and reduced expression of proteins involved in mitochondrial biogenesis, such as PGC-1α (peroxisome proliferator-activated receptor-gamma coactivator 1-α) and SIRT3 (Sirtuin 3) [41]. Alterations in mitochondrial content may help to increase bioenergetic efficiency under adverse conditions [43]. mtDNA copy number has been inversely correlated with gestational age and birth weight [45]. In pregnancies complicated by placental insufficiency, compared to controls, mtDNA levels were increased [40], such as those with IUGR [5,46]; an inverse relationship was found between the mtDNA content and the level of oxygen in the placenta (umbilical venous pO_2_) [46]. Increased mtDNA content could represent a compensatory mechanism for hypoxia, as, when oxygen becomes limited, modulation of the mitochondrial function plays an important role in general biological adaptation [5,46].

In addition to the reduced ATP production capacity, impaired mitochondrial function may lead to a limited supply of TCA metabolites (e.g., NAD+, α–KG, and AcCoA), which are used for ATP synthesis and supporting cell survival, rather than for other processes (e.g., epigenetic processes), forcing changes in growth and physiology in the developing embryo. Diminished mtDNA turns into alterations in one-carbon (1-C) metabolism and TCA activity, not only affecting gene transcription, but also potentially modifying methionine metabolism, which may impact methylation [6]. The understanding of mitochondrial function on epigenetics is a recently recognized research topic, and three main mechanisms regulating gene expression within the mitochondria have been described: DNA methylation, non-coding RNAs, and post-translational modifications of nucleoid-associated proteins [47].

## 4. Nutrients Involved in Mitochondrial Function During Pregnancy

Nutritional programming refers to the processes of physiological–functional adaptations or morphological changes in the offspring due to early exposure to nutrient stimuli. This implies diverse long-term consequences and depends on factors such as the type of nutrient, the developmental stage of the stimuli, length and extent of exposure, and sex, among others [1]. Antenatal nutrition plays a major role in the offspring’s susceptibility to disease, which may be influenced by the macronutrient and micronutrient balance. The availability of nutrients regulates the activity of metabolic pathways and mitochondrial metabolism. Disturbances in nutrient flux may drastically affect long-term development, through the regulation of inheritable changes in the epigenome prior to differentiation [6]. A healthy and nutritionally adequate lifestyle should promote balanced energy supply-demand in agreement with mitochondrial fusion, fission, mitophagy, and biogenesis, which leads to adequate functionality of the mitochondrial network and to the integrity of the mitochondrial genome [48].

**Figure 1:**
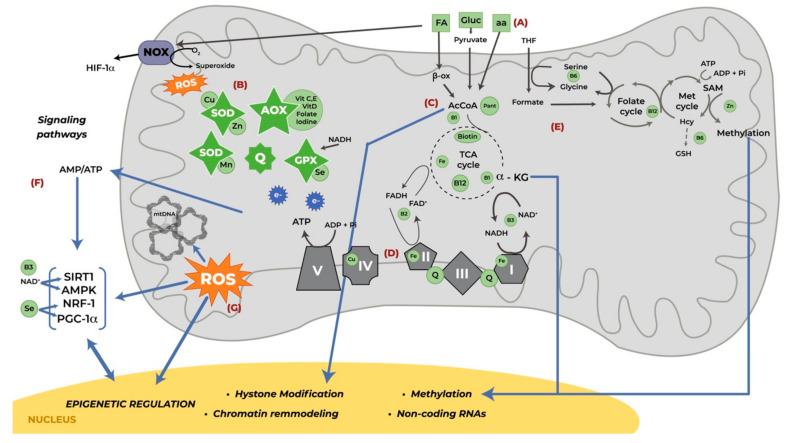
Nutrients involved in mitochondrial function which may influence perinatal outcomes. (**A**) Excessive substrate consumption could increase ROS production. (**B**) Micronutrients are part of the antioxidant system. (**C**) The TCA cycle requires micronutrients for the supply of metabolites for biosynthetic pathways and for transcriptional and epigenetic processes. (**D**) ATP production by the ETC complexes involves micronutrients as cofactors and antioxidants. (**E**) Mitochondrial production of formate is used in 1-C metabolism for methylation, requiring vitamins and minerals. (**F**) Micronutrients participate in the activation of signaling pathways involved in mitochondrial function, as well as influence epigenetic mechanisms. (**G**) Excessive ROS lead to oxidative damage, inflammation, altered mitochondrial function and epigenetic regulation. Abbreviations: I-V, mitochondrial complexes I–V; α–KG, α-ketoglutarate; β-ox, β-oxidation; aa, amino acids; AcCoA, acetyl coenzyme A; ADP, adenosine diphosphate; AMP, adenosine monophosphate; AMPK, cyclic adenosine monophosphate activated protein kinase; AOX, antioxidant system; ATP, adenosine triphosphate; Cu, copper; FA, fatty acids; FAD+/FADH, oxidized/reduced flavin adenine dinucleotide; Fe, Iron; Gluc, glucose; GPX, glutathione peroxidase; GSH, glutathione; Hcy, homocysteine; HIF-1α, hypoxia-inducible factor 1α; mt, mitochondrial; NAD+/NADH, oxidized/reduced nicotinamide adenine dinucleotide; NOX, NADPH oxidase; NRF-1, Nuclear Respiration Factor 1; Pant, pantothenic acid; PGC-1α, peroxisome proliferator-activated receptor-gamma coactivator 1-α; Pi, phosphate; Q, coenzyme Q10; ROS, reactive oxygen species; SAM, S-adenosylmethionine; Se, selenium; SIRT1, sirtuin 1; SOD, superoxide dismutase; TCA, tricarboxylic acid; Vit, vitamin; Zn, zinc.

Different nutritional elements are essential for mitochondrial function and optimizing their availability serves to improve the outcome of clinical imbalances. Mitochondrial bioenergetic performance is optimal when substrates and cofactors are available in adequate amounts and proportions [20]. Some of the central mitochondrial functions are maintaining an efficient energy supply, providing an optimal redox environment, and signaling. For these, mitochondria metabolize oxygen and oxidizable substrates, having a continuous challenge in providing energy while maintaining ROS within physiological levels compatible with “healthy” signaling [48]. Figure 1 shows the multiple nutrients involved in the diverse mitochondrial metabolic activities, which are detailed in the following sections.

Animal studies have shown associations between diet during pregnancy and changes in oxidative stress markers and mitochondrial damage, indicating that mitochondrial dysfunction may be a consequence of an altered nutritional environment during early life [49]. A 50% energy restriction in sheep during the third trimester of pregnancy causes a decrease in mitochondrial function in muscle fibers in adult offspring, as evidenced by the reduction of mitochondrial VO_2max_, a lower respiratory coupling ratio, and an increase in the expression of PGC-1α [50]. In pigs, an energy-restrictive diet during pregnancy (approximately 13% restriction) decreased mtDNA in fetal skeletal muscle, as well as diminished the expression of PGC-1α, sirtuin 1 (SIRT1), NRF-1α (Nuclear Respiration Factor-1α), TFAM, and the β subunit of mitochondrial ATP synthase, when compared with fetuses of mothers with a standard diet during pregnancy [51].

Some possible mechanisms underlying nutritional programming where mitochondrial function is involved include nutrient-sensing signals, oxidative stress, and epigenetic regulation, among others [1]:

Nutrient-sensing signals: Maternal nutritional status determines fetal development and metabolism by means of nutrient-sensing signals. Some of these signals, reflecting the importance of mitochondrial function, are: SIRT1 (regulates mitochondrial biogenesis and ROS production), cyclic adenosine monophosphate activated protein kinase (AMPK), and peroxisome proliferator-activated receptors (PPAR) [1]. Different metabolic diseases have been related to impaired nutrient-sensing signals. With insufficient nutrients, AMPK and SIRT1 are stimulated (by rises in intracellular levels of AMP and NAD+, respectively), and their interaction regulates PPARs and their target genes, programming disease later in life. A maternal diet excessive in fat increases PPAR expression and fetal fat mass, decreasing SIRT1 expression. Pharmacological regulation of AMPK or PPAR signaling could prevent hypertension and metabolic syndrome as shown in fetal programming models [1].

Epigenetic regulation: Substrate availability regulates the crosstalk between mitochondrial function and the epigenome and is mediated by energy as well as redox metabolites. The equilibrium between substrate availability and requirement determines the mitochondrial supply of intermediaries, affecting the epigenome. The nutrient composition of the diet defines the availability of substrates, cofactors, and effectors (ATP, AcCoA, NADH, and α-KG), thus generating patterns of epigenetic modifications through methylation, acetylation, or oxidation, which regulate signal transduction pathways [48].

Oxidative stress: In the intrauterine environment, there are various conditions that favor oxidative stress, among which the large number of mitochondria present in the placenta and its high vascularity have been recognized, which is why it is exposed to high oxygen content and maternal partial pressure, thus increasing the production of superoxide. Reactive oxygen species participate in differentiation processes [52]. Conditions such as obesity or excess energy consumption have been associated with the exacerbation of ROS production [17,36]. In an intrauterine environment with excessive production of ROS, oxidative stress will cause damage, affecting vulnerable organs in fetal development, as the embryo and the fetus have poor antioxidant capacity [1]. Overfeeding, excessive intake of carbohydrates, animal proteins, and saturated fat induce ROS formation and oxidative stress, which may contribute to the development of metabolic disorders, in particular insulin resistance. Excessive energy consumption and/or obesity have been associated with a pro-oxidant environment and increased oxidative damage [19]. During gestation, numerous nutritional insults such as calorie restriction, high fructose diet, low protein diet, high fat diet, and zinc and iron deficiency, as well as a low methyl-donor diet, have been related to oxidative stress damage and repercussions in adult offspring [1].

### 4.1. Macronutrients

All major substrates derived from dietary intake (e.g., glucose, lipids, and amino acids) go through mitochondrial metabolism, resulting in readily available metabolites such as ATP, AcCoA, NADH, and ROS, which, in turn, have the potential to alter the metabolism–epigenome–genome axis, ROS production, de novo lipogenesis, β-oxidation, insulin resistance, and subsequent comorbidities [48]. Diet-derived mitochondrial metabolites together with S-adenosylmethionine (SAM), the primary methyl donor, promote epigenetic modifications. For instance, the phosphorylation of nuclear and cytoplasmic signal transduction proteins and histone tails is ATP mediated; AcCoA donates acetyl groups for the acetylation of chromatin and signal transduction proteins, modifying DNA transcription and replication; NAD+ is a cofactor of sirtuins to deacetylate proteins; and DNA is methylated through SAM [48].

Excessive calorie consumption causes more substrates to enter mitochondrial OXPHOS, increasing the number of electrons donated to the ETC as well as the amount of ROS [17]. ROS produced in mitochondria contribute to mitochondrial damage, which not only affects cellular signaling but also causes a range of dysfunctions comprising metabolic disorders [53,54]. An excess in free fatty acids and/or glucose increases the production of AcCoA, which promotes the synthesis of NADH. Increased availability of NADH stimulates the generation of electrons by the mitochondrial complex I and increases membrane potential (hyperpolarization), to the point where complex III ceases activity, causing an extended life for coenzyme Q10/ubiquinone (CoQ10). Higher CoQ10 prompts superoxide from oxygen reduction. Superoxide is converted to hydrogen peroxide in the mitochondria, which in turn can produce the highly reactive hydroxyl radical [54]. Although superoxide and hydrogen peroxide have physiological functions such as immune defense (autophagy and macrophage oxidative burst), apoptosis, stem cell differentiation, and activation of antioxidant defense by activating Nrf2, among others [55,56], the redox state is altered when there is an excessive production (e.g., with excessive substrates) and the antioxidant systems become overwhelmed, leading to oxidative damage with the potential to oxidize mitochondrial proteins, DNA, and lipids, as well as magnifying superoxide-initiated oxidative stress. The excessive generation of ROS can activate transcription factors and lead to many downstream effects, including the activation of inflammatory cascades and even more free radical production [54]. Postprandial oxidative stress leads to inflammation, mainly mediated by Nuclear Factor Kappa B (NF-κB); the release of inflammatory cytokines such as Tumor Necrosis Factor-α (TNF-α) and Interleukin (IL-6), and acute phase reactants such as C-reactive protein (PCR), which are involved in the most frequent pathways associated with food consumption and inflammation in humans. Chronic excessive caloric (e.g., free fatty acids and glucose) intake results in obesity, which induces persistent states of inflammation as a consequence of proinflammatory factors secreted by white adipose tissue [17,54].

Postprandial hyperglycemia, through different mechanisms, can induce inflammatory cascades as mediated by an increase in oxidative stress, probably initiated by mitochondrial glucose overload. Various mechanisms have been suggested for hyperglycemia-induced oxidative stress. During hyperglycemia, the polyol pathway is induced, where aldose reductase depletes NADPH to convert excess glucose to sorbitol. Glutathione reductase uses NADPH, such that the scarcity of the substrate leads to reduced levels of GSH, a pivotal element of the antioxidant system, promoting susceptibility to oxidative stress damage [22].

Diets with a high content of fat (35–60% of total calories) have been associated with increased body weight, insulin resistance, deposition of fat in various organs, and development of a hypoxic status in the fat-depositing organs [19]. A high fat diet will increase the synthesis of ATP, activating the usage of mitochondrial ETC and oxygen, resulting in acute hypoxic events where superoxide bursts can occur [57,58]. With this type of diet, HIF-1α is induced, contributing to compensation of the chronic inflammatory response of adipose tissue [19]. Excessive ROS production has been observed in mitochondria from skeletal muscle, kidney, liver, and adipose tissue from obese animals fed with excess lipids. The plasma membrane contains NADPH oxidase (NOX), which turns oxygen to superoxide [59] and may be involved in the generation of nutrient-based ROS. Increased fatty acids (e.g., from overfeeding or obesity) can activate NOX in fat cells or in other cells, and induce or aggravate ROS production [17,19,22].

Maternal high-fat diets have been associated with insulin signaling, brain appetite regulation, immune function, blood pressure, aortic structure, kidney function, plasma lipids, and antioxidant defense in the offspring [60,61]. A reduction in liver mtDNA was found in adult rats born from mothers fed a high-fat diet during pregnancy and lactation, regardless of sex or diet of the offspring. Furthermore, the mtDNA copy number in the offspring liver was significantly associated with fatty liver [49]. Another study found that the offspring of rats fed a high-fat diet revealed reprogramming of pathways linked to the immune response, inflammation, OXPHOS, and mitochondrial function, as well as reduced protein expression of mitochondrial OXPHOS complexes. In addition, higher perirenal and abdominal fat and plasma insulin concentrations were found in the offspring of mothers with a high-fat diet [60].

Fetal exposure to protein restriction could alter the levels of mtDNA methylation of newborns and modulate their mitochondrial OXPHOS capacity, resulting in long-term mitochondrial dysfunction that could promote the development of metabolic disease [21,47]. Decreased glucose tolerance and elevated blood pressure were found in adult offspring of rats fed a low protein diet during pregnancy, caused by fetal programming of arterial dysfunction and abnormal pancreatic development. Mitochondrial dysfunction has been shown to be consistent with these metabolic disorders, where excessive ROS seems to be the underlying mechanism [50]. In piglets, antenatal protein deficiency impairs offspring mtDNA methylation and decreases (tendency) hepatic mtDNA copy number; only males showed a significant reduction in mtDNA [62]. 

### 4.2. Micronutrients

Mitochondria need a variety of cofactors for optimal function, among which various micronutrients are essential. Vitamins and minerals act either as cofactors in energy metabolism and/or as antioxidants; these two functions are interconnected, considering that antioxidants may prevent injury to the energy metabolism enzymes, protecting from a decline in energy production [20]. The central antioxidant micronutrients are copper, zinc, manganese, selenium, vitamins (E, C and A), and the glutathione system [1]. Dietary and enzymatic antioxidants interact with each other to control the production of ROS and protect cells from damage; deficiencies of proteins (proteins provide the amino acids needed for the synthesis of antioxidant enzymes, such as glycine, glutamate, and cysteine for GSH [63]), selenium, and zinc are associated with cell injury [8,20]. The antioxidant system protects against mitochondrial damage by neutralizing free radicals, sequestering transition metal ions, restoring damaged molecules, and interrupting ROS-initiated damage chain reactions [20]. Furthermore, many micronutrients are key pieces of the active site of antioxidant enzymes or pact as cofactors in their regulation [8]. A recent meta-analysis (2953 cases and 3621 controls) showed a significant reduction in total antioxidant capacity, SOD, GSH, and vitamins E and C in pre-eclamptic women [64].

Redox imbalances associated with antioxidant micronutrients may offer a mechanistic explanation of the effects on fetal programming [8]. Copper deficiency may directly affect cuproproteins, such as SOD, which also requires zinc for its catalytic activity (Cu/Zn SOD) [7,8]. Cu/Zn SOD and manganese SOD are part of the defense antioxidants in the placenta [34], providing protection during fetal development. Copper is also required for mitochondrial OXPHOS, for the activity of the mitochondrial cytochrome c oxidase enzyme [7,8]. Increased ROS levels and copper deficiency diminishes SOD activity and increases peroxynitrite formation, which induces the oxidation of lipids and DNA [65,66,67]. During pregnancy, low copper has been implicated in defects in fetal development, affecting the central nervous system, cardiovascular, and skeletal systems, leading to poor immunocompetence and behavioral abnormalities in offspring [8]. Low copper is linked to pregnancy-induced hypertension [68] and positively correlated with neonatal weight [69].

Maternal malnutrition has been associated with multiple micronutrient deficiencies, a major cause of IUGR and miscarriage, which are frequent in developing countries. Low serum zinc levels, as well as copper and manganese, have been associated with infertility and suboptimal pregnancy outcomes, such as placental abruption, pregnancy-induced hypertension, premature rupture of membranes, prolonged labor, atony postpartum hemorrhage, preterm labor, and low birth weight [8,70]. Zinc participates in carbohydrate and protein metabolism, nucleic acid synthesis, and plays an important role as a cofactor of antioxidant enzymes and induces metallothionein, which reduces hydroxyl radicals. It also prevents DNA strand breakdown and promotes DNA repair [7,34]. Many histone deacetylases are zinc-dependent metalloenzymes [71]. Mild to moderate deficiency of zinc may be relatively common throughout the world [70]. A meta-analysis showed that zinc supplementation resulted in a 14% reduction in preterm birth compared with placebo, but this result came from trials involving women in low-income countries with high perinatal mortality [70].

Selenium is the cofactor of many selenoproteins, including the GPX antioxidant enzymes, thioredoxin reductases, and selenoprotein-P, which are all part of the placental antioxidant defense [34]. Selenium is also involved in mitochondrial biogenesis, stimulating PGC-1α and NRF-1 [7,20]. Supplementation with selenium significantly improves mitochondrial respiration and increases mitochondrial content in trophoblasts [72]. A systematic review and meta-analysis showed an inverse association between the concentration of selenium and the risk of PE, where selenium supplementation significantly reduced the incidence of PE [73] by reducing the oxidative stress [74]. Adequate maternal serum selenium could reduce the risk of IUGR by 11% and the risk of premature birth (<34 weeks) by 7% [75]. Women with low selenium have about three times higher risk of having a small for gestational age infant [76].

The diverse functions of iodine include bactericidal activity and apoptosis induction, and it is involved in trophoblast migration, invasion, and differentiation. As an antioxidant, iodine may act directly as an electron donor and compete for binding sites with free radicals [77]. Iodine deficiency remains a significant health problem worldwide and affects both industrialized and developing nations [78]. Mild iodine deficiency has been associated with increased oxidative stress and decreased total antioxidant capacity and SOD activity in pregnant women in Mexico [79]. It has also been reported that 70% of women with hypertensive disease in pregnancy were iodine deficient [77]. Mild iodine deficiency was an independent risk factor for gestational diabetes mellitus [80].

Vitamin E is an indispensable micronutrient for the development of the placental labyrinth trophoblast. Low α-tocopherol blood concentrations are present in abnormal pregnancies, which could reflect that vitamin E requirements increase throughout pregnancy [8]. Vitamin C protects mtDNA from oxidative damage by ROS [7]. Vitamin C is a free radical scavenger which is effective in regenerating the antioxidant form of vitamin E [81]. Vitamin C and vitamin E supplementation have been associated with a reduction of lipid peroxidation in pregnant women [82]. Despite the overwhelming evidence that oxidative stress plays a role in PE, supplementation of both vitamins is not conclusive for PE prevention [81,82,83].

Vitamin D supplementation in pregnant women results in a significant increase in total antioxidant capacity and total GSH concentrations, as well as a significant decrease in serum PCR compared to placebo. It also decreases plasma glucose, as well as systolic and diastolic blood pressure [84]. In addition, vitamin D supplementation during pregnancy and lactation affects DNA methylation in both mothers and their infants [85].

Folic acid plays a central role in 1-C metabolism, synthesis, and repairing DNA, as well as having antioxidant functions. It might be an important micronutrient for the maintenance of mitochondrial function. Folic acid deficiency has been associated with an increase in ROS and oxidative damage, altered antioxidant enzymatic activities, decreases in mtDNA biogenesis, and mitochondrial oxidative deterioration. In addition, increased intake of dietary folic acid may protect against mtDNA deletion cumulative damage. Hepatic mtDNA is lower in piglets with IUGR from mothers without folic acid supplementation and present a higher concentration of malondialdehyde (a lipid peroxidation product) and lower SOD activity. No difference was reported in mtDNA between IUGR piglets from the supplemented mothers and normal birthweight piglets. Expression of PGC-1α and TFAM were reduced in piglets with IUGR, while maternal folic acid supplementation prevented this effect, to some extent [86].

There is a connection between mitochondrial activity and methylation, due to the mitochondrial synthesis of formate, the folate cycle, and 1-C metabolism [6]. The 1-C metabolism provides methyl groups for methylation and the development of fetal DNA, but also one-carbon groups for at least 50 different methylation reactions (proteins, phospholipids, and nucleic acids), as well as for the synthesis of purines, thymidylate, creatine, phosphatidylcholine, and multiple hormones [87,88,89]. Mitochondria is the main site of formate production. Formate enters the folate cycle and is used to provide methyl groups for the re-methylation of homocysteine to methionine and further SAM production, or for purine biosynthesis or thymidylate synthesis. Formate metabolism is highly active in fetuses and in the placenta of pregnant rats [90]. Genetic mouse models with impaired mitochondrial formate metabolism showed an elevated incidence of neural tube diseases [91]. This suggests that formate could play a critical role in embryonic development [92]. Metabolism of 1-C involves nutrient availability, amino acid metabolism, and mitochondrial activity; any deviation in these could alter the epigenome [6].

Vitamins B12, B6, B2, choline, methionine, and SAM are crucial to 1-C metabolism. DNA methyltransferases use SAM, which involves members of the zinc family, methionine, and B vitamins. SAM, the reactive methyl carrier, is important in the establishment and maintenance of the epigenome [93]. Mitochondrial synthesis of ATP and folate modulate SAM production [48]. Maternal concentrations of these biomarkers are related to changes in methylation. Maternal folic acid supplementation directly affects the methylation status of the IGF-2 gene (Insulin-like growth factor-2) in babies up to 17 months of age, presenting 4.5% higher methylation. IGF-2 methylation has also been associated with SAM blood levels in the mother but not in the child [94]. A meta-analysis of wide-epigenome studies showed an inverse association of 48 CpG sites with maternal plasma folate concentrations during pregnancy [95]. Pregnant women with low B12 show increased expression of adipogenic and lipogenic genes, which may mediate an adipogenic and insulin resistance phenotype, leading to obesity [96]. Higher maternal B6 concentrations have been positively associated with offspring DNA methylation levels at the MEG3, a differentially methylated region known to be involved in fetal growth and development [97], possibly affecting birth weight, growth, and cardiometabolic risk later in life [98]. The concentration of methyl donor nutrients in maternal blood predicts methylation at metastable epialleles in DNA extracted from their infants postnatally [99]. Pregnant women with the higher dietary methyl-group intake (folate, betaine, choline, and methionine) showed higher global DNA methylation in the third trimester [100]. Inadequate maternal blood concentrations of some methyl-donor nutrients have been associated with a decrease in overall DNA methylation and a higher birth weight [101]. Hypo- or hyper-methylation of DNA could be associated with different neonatal and childhood diseases [102]. Supplementation with these methyl-donor nutrients during gestation has been associated with better neurodevelopment outcomes in infants [103,104].

Other B vitamins are used to support OXPHOS (vitamins B3 and B2), metabolic pathways (vitamin B1, choline, and folate), and promote mitochondrial biogenesis (vitamin B3) [105]. Riboflavin (vitamin B2) is necessary for the function of complexes I and II in the mitochondria [7]. Derived from vitamin B2, flavin adenine dinucleotide (FAD) is generated by the mitochondria and functions as a prosthetic group for enzymes with redox activity. The nuclear located LSD1 (Lysine-Specific Demethylase-1), which regulates mitochondrial respiration and energy expenditure, uses FAD [48]. Riboflavin is a common intervention to improve ETC efficiency in primary mitochondrial disorders. Thiamine (vitamin B1) is a cofactor of α-ketoacid dehydrogenase complexes including pyruvate dehydrogenase complex (PDHc), α-KG dehydrogenase (α-KGDH), and branched-chain α-ketoacid dehydrogenase [105]. Pantothenic Acid is a precursor of AcCoA and, therefore, is crucial in the function of PDH and α-KGDH. Vitamin B12 is necessary for the synthesis of succinyl CoA from methylmalonyl-CoA and, due to its participation in the regulation of NF-kB by inhibiting inducible nitric oxide synthase, it has antioxidant and anti-inflammatory functions [20].

The different forms of vitamin B3 (niacin, niacinamide, nicotinic acid, and nicotinamide), along with tryptophan, are biosynthetic precursors to NAD [106]. During the development of the blastocysts, the production of NAD+ increases (by converting pyruvate to lactate) to ensure glycolysis. NADH is transported through the malate aspartate transporter (MAS), in the inner mitochondrial membrane, to maintain the NAD+:NADH ratio. During embryonic development, oxidative metabolism depends on MAS activity and its inhibition alters embryonic metabolism, ATP production, and decreases blastocyst development and placental and fetal growth. This underlines the importance of NAD+ in fetal programming, through coordinated communication between the cytoplasm and the mitochondria, to sustain optimal mitochondrial function and ensure adequate development [6,48]. NAD also participates in PPAR activation and sirtuin activity, which are involved in modulating cell metabolism, mitochondrial biogenesis, cell survival, DNA repair, and ROS production [6,48,105].

Biotin is essential for normal mitochondrial and cellular function [107], and evidence suggests that biotin may affect gene expression, as it is attached to histones [108]. Marginal biotin deficiency could be a frequent occurrence in the first trimester, especially in women without supplementation [109]. There is some concern that biotin deficiency could cause human birth defects, as it has been shown to be teratogenic in mice [110]. Biotin is a coenzyme for five mitochondrial carboxylases [20], and biotin insufficiency decreases its activity, causing a decrease in two precursors of heme groups (mitochondrial succinyl-CoA and glycine), having heme group deficiency as a consequence. The synthesis of heme groups in mitochondria requires biotin, but also pyridoxine, pantothenate, zinc, riboflavin, iron, and copper. The inadequacy of any of these micronutrients could lead to a deficiency of heme groups [107]. Heme is an essential prosthetic group and a crucial cofactor in various biological processes—especially during pregnancy for fetal growth—such as oxygen transport (heme groups are components of hemoglobin) and storage (also components of myoglobin) and electron transfer (part of respiratory cytochromes) [111]. Iron, which is also required for oxygen transport and storage, is additionally important in cellular bioenergetics. Iron is part of iron–sulfur groups, the primary goal of which is electron transfer in the ETC complexes, as well as having a role in the function of various enzymes in the Krebs cycle [112]. An increase in maternal serum iron reduced the risk of PE by 27% [75].

CoQ10 is an endogenously synthesized lipid that aids in preserving the mitochondrial inner membrane and stability of the OXPHOS complex. It transports electrons to complex III from complexes I and II. CoQ10 is a ROS scavenger; in the form of ubiquinol, it inhibits lipid peroxidation and oxidative damage in mitochondrial inner membrane proteins and in mtDNA. CoQ10 deficiency results in impaired activity of mitochondrial complexes and in a decrease in ATP production [20,105]. Women with PE have lower plasma levels and higher placental levels of CoQ10 compared to healthy pregnant women [113,114]. CoQ10 supplementation improved the function of the mitochondria in the placenta, as well as the signs of PE in rats, showing a significantly higher level of mitochondria membrane potential, lower systolic pressure, lower 24 h urine protein, and greater weight of the offspring [114].

Recently, Prilliani et al. observed that mtDNA increases from the beginning of pregnancy towards the end, independent of the type of supplementation administered (multiple micronutrients or iron + folic acid), which could suggest a compensatory mechanism to meet increasing energy requirements and overcome the rise in oxidative stress during pregnancy. They found that, with multiple supplementation, the mtDNA increase was lessened (relatively small difference), hypothesizing that mixed supplementation could mitigate the change in mtDNA copy number by supporting more efficient mitochondrial function, protecting against oxidative damage and preserving mitochondria and the quality of mtDNA [7].

## 5. Conclusions

The relevance of mitochondria clearly extends beyond energy production for the cell. The functionality of the mitochondria affects multiple signaling pathways, cell proliferation, and changes in gene expression involved in determining cell fate. They play a critical role in the physiological changes during pregnancy, regulating embryogenesis, embryonic implantation, and fetoplacental development. Due to the high energy and micronutrient demand, fetal development requires optimal mitochondrial activity; therefore, nutritional imbalances may have a negative impact on all these processes, programming the baby for long-term risk of metabolic disease.

Diet has a major impact on mitochondrial function, where excess energy and insufficient micronutrients could have detrimental consequences on both maternal and perinatal health. As a consequence, it is important to optimize metabolic function by meeting the dietary requirements of energy, vitamins, and minerals during pregnancy. Dietary micronutrient deficiencies are highly prevalent, particularly in low-income countries [115]. The main dietary sources of energy come from industrialized and highly processing foods (i.e., sugary drinks, snacks, desserts, and sweet cereals) leading to excessive consumption of energy, carbohydrates, and lipids, while the consumption of healthy foods (i.e., vegetables, fruits, and legumes) is low, leading to low micronutrient intake; these factors may impair mitochondrial function. These dietary aspects have been related to the presence of important metabolic disorders that could deeply affect pregnancy and fetal programming.

With the burden of perinatal complications, diet is frequently placed last, giving priority to other strategies such as medications, limiting the beneficial downstream effects provided by a healthy diet. Standard care during pregnancy should emphasize the importance of optimizing nutritional status and promoting the adoption of good eating and lifestyle habits. Reducing the energy density of the diet and increasing the consumption of natural, fresh foods that provide high micronutrient density, as well as individualized nutrient supplementation during pregnancy, may be beneficial. Early access to prenatal care should be ensured to receive adequate nutrition care.

As nutrient availability regulates mitochondrial function, there is a potential opportunity for mitochondria to be a target for the prevention/treatment of pregnancy complications through diet and supplementation. A greater understanding of what type of diet or supplementation scheme (dosage and timeframe) is necessary to optimize the mitochondrial function and achieve the maximum potential for fetal development in the general population, as well as in undernourished groups or in women at high risk of adverse perinatal outcomes. 

Mitochondrial integrity, communication, and metabolism are of utmost importance, due to their crucial role in meeting the fetal energy demand for growth and development; influence on different biosynthetic pathways, such as those involving cholesterol, lipids, nucleic acid, and heme groups; active role in one-carbon metabolism and methylation; and importance in terms of the availability of metabolic co-factors that modulate epigenetic processes capable of establishing significant short- and long-term effects on maternal and infant health. Understanding the complexity of the diverse roles of mitochondria during pregnancy is of particular significance for perinatal health and could provide insight into individual responses to diet, lifestyle, medication, and other environmental exposures.

## Abbreviations

1-C	One-carbon
α–KG	α-ketoglutarate
α-KGDH	α-KG dehydrogenase
AcCoA	Acetyl coenzyme A
AMP	Adenosine monophosphate
AMPK	Cyclic adenosine monophosphate activated protein kinase
ATP	Adenosine triphosphate
CoQ10	Coenzyme Q10/Ubiquinone
ETC	Electron transport chain
FAD	Flavin adenine dinucleotide
GSH	Glutathione
GPX	Glutathione peroxidase
HIF-1α	Hypoxia-inducible factor 1α
IGF-2	Insulin-like growth factor-2
IL	Interleukin
IUGR	Intrauterine growth restriction
MAS	Malate aspartate transporter
mtDNA	Mitochondrial DNA
NAD/NADH	Oxidized/Reduced nicotinamide adenine dinucleotide
NCD	Non-communicable diseases
NF-κB	Nuclear Factor Kappa B
NOX	NADPH oxidase
NRF-1α	Nuclear Respiration Factor-1α
O2	Oxygen
OXPHOS	Oxidative phosphorylation
PCR	C-Reactive protein
PDHc	Dehydrogenase complex
PE	Preeclampsia
PGC-1α	Peroxisome proliferator-activated receptor-gamma coactivator 1-α
PPAR	Peroxisome proliferator-activated receptors
ROS	Reactive oxygen species
SAM	S-adenosylmethionine
SIRT	Sirtuin
SOD	Superoxide dismutase
TCA	Tricarboxylic Acid cycle
TFAM	Mitochondrial Transcription Factor A
TNF-α	Tumor Necrosis Factor-α
